# Supplementary material for: Artificial intelligence as a surrogate brain: bridging neural dynamical models and data
Source: Natl Sci Rev. 2025 Oct 25;13(3):nwaf457. doi: 10.1093/nsr/nwaf457 (PMC12866659; doi:10.1093/nsr/nwaf457)
Supplement: nwaf457_Supplemental_File [file nwaf457_supplemental_file.pdf]

Supplementary

Contents

1 BENCHMARK AND EVALUATION **2**
- 1.1 Evaluation Metric . . . 2
  - 1.1.1 Error metrics in state space . . . 2
  - 1.1.2 Metrics in probability space . . . 3
  - 1.1.3 Goodness of fit . . . 3
  - 1.1.4 Functional similarity . . . 3
  - 1.1.5 Topological similarity . . . 4
- 1.2 Examples of Benchmark . . . 4

2 MATHEMATICAL BACKGROUND KNOWLEDGE **6**
- 2.1 Well-posedness of Inverse Problem . . . 6
  - 2.1.1 Basic Setup . . . 6
  - 2.1.2 Three Conditions for Well-posedness . . . 6
- 2.2 Introduction to Optimization Methods . . . 7

3 SUPPLEMENTARY TABLE **9**
- 3.1 Neural Mass Model . . . 9
- 3.2 Comparison of White-, Black-, and Grey-box Approaches . . . 10

1 BENCHMARK AND EVALUATION

Due to the limited number of AI-based surrogate brain models currently available, and the absence of widely recognized and influential benchmarks or software, we propose a straightforward training and evaluation workflow for AI models in surrogate brain research, The dataset used in this study is the publicly available HUP iEEG dataset from OpenNeuro [1, 2]. The related code can be found here: `https://github.com/yinuo-zzz15/review_surrogate_brain.git`.

We evaluate four base models as surrogate brain models: basic RNN, low-rank RNN [3], EI RNN [4], and dendritic PLRNN [5]. For model evaluation, we provide a comprehensive set of 10 evaluation metrics, including Mean Squared Error (MSE), Mean Absolute Error (MAE), Kullback-Leibler divergence (KL Divergence), Hellinger distance, explained variance (EV), R^2, spectral similarity, functional connectivity similarity, H1 Wasserstein distance for persistent homology, and fractal dimension similarity. These metrics cover five key aspects: error-based metrics, probability distribution metrics, goodness of fit, functional similarity, and topological similarity, enabling a holistic evaluation of surrogate brain models.

It is important to note that evaluations related to behavior/decoding typically need to be coupled with specific tasks. Here, we primarily introduce evaluation functions focusing on signal fidelity.

1.1 Evaluation Metric

We use 10 evaluation metrics to assess the performance of the surrogate brain models. Below are the mathematical formulations for each metric, where x represents the true data and $\hat{x}$ represents the results generated by the surrogate brain model.

1.1.1 Error metrics in state space

Mean Squared Error (MSE):. The MSE measures the average squared difference between the true values and the predicted values:

$$\text{MSE} = \frac{1}{N}\sum_{i=1}^{N}(x_i - \hat{x}_i)^2 \tag{1}$$

where x_i is the true value and $\hat{x}_i$ is the predicted value at the i-th time point, and N is the total number of time points.

Mean Absolute Error (MAE): The MAE measures the average absolute difference between the true values and the predicted values:

$$\text{MAE} = \frac{1}{N}\sum_{i=1}^{N}|x_i - \hat{x}_i| \tag{2}$$

1.1.2 Metrics in probability space

Kullback-Leibler Divergence (KL Divergence): The KL divergence measures the difference between two probability distributions. For normalized true values x and predicted values $\hat{x}$, the KL divergence is given by:

$$D_{\mathrm{KL}}(x \parallel \hat{x}) = \sum_{i=1}^{N} x_i \log \frac{x_i}{\hat{x}_i} \tag{3}$$

Hellinger Distance: The Hellinger distance quantifies the similarity between two probability distributions. It is given by:

$$H^2(x, \hat{x}) = \frac{1}{\sqrt{2}} \sum_{i=1}^{N} \left(\sqrt{x_i} - \sqrt{\hat{x}_i} \right)^2 \tag{4}$$

1.1.3 Goodness of fit

Explained Variance (EV): The explained variance measures the proportion of variance in the data that is explained by the model:

$$\mathrm{EV} = 1 - \frac{\sum_{i=1}^{N} (x_i - \hat{x}_i)^2}{\sum_{i=1}^{N} (x_i - \bar{x})^2} \tag{5}$$

where $\bar{x}$ is the mean of the true values x.

R-squared (R^2): The R^2 value represents the proportion of variance explained by the model. It is given by:

$$R^2 = 1 - \frac{\sum_{i=1}^{N} (x_i - \hat{x}_i)^2}{\sum_{i=1}^{N} (x_i - \bar{x})^2} \tag{6}$$

1.1.4 Functional similarity

Spectral Similarity: Spectral similarity measures the similarity of the signals in the frequency domain, and is defined as:

$$\text{Spectral Similarity} = \frac{\left| \sum_{i=1}^{N} \left(\hat{X}_{\text{true}}(f_i) \hat{X}^*_{\text{pred}}(f_i) \right) \right|^2}{\sum_{i=1}^{N} \left| \hat{X}_{\text{true}}(f_i) \right|^2 \sum_{i=1}^{N} \left| \hat{X}_{\text{pred}}(f_i) \right|^2} \tag{7}$$

where $\hat{X}(f_i)$ is the Fourier transform of the signal, and $*$ denotes the complex conjugate.

Functional Connectivity Similarity: Functional connectivity similarity is quantified by calculating the correlation between the true and predicted signals:

$$\text{Functional Connectivity Similarity} = \frac{\sum_{i=1}^{N}(x_i - \bar{x})(\hat{x}_i - \bar{\hat{x}})}{\sqrt{\sum_{i=1}^{N}(x_i - \bar{x})^2 \sum_{i=1}^{N}(\hat{x}_i - \bar{\hat{x}})^2}} \tag{8}$$

where $\bar{x}$ and $\bar{\hat{x}}$ are the means of the true and predicted values, respectively.

1.1.5 Topological similarity

Persistent Homology Wasserstein Distance: The Wasserstein distance is used to measure the difference between the persistent homology diagrams of the true and predicted signals. The distance is given by:

$$\text{Wasserstein Distance} = \min \sum_{i} \left| \text{persistence diagram}_{\text{true}}(i) - \text{persistence diagram}_{\text{pred}}(i) \right| \tag{9}$$

Fractal Dimension Similarity: The fractal dimension measures the complexity of a signal and can be calculated using the box-counting method or recursive formulas. The general expression is:

$$\text{Fractal Dimension} = \lim_{\epsilon \to 0} \frac{\log N(\epsilon)}{\log(1/\epsilon)} \tag{10}$$

where $N(\epsilon)$ is the number of boxes required to cover the signal at scale ϵ.

1.2 Examples of Benchmark

We conducted experiments to investigate the performance of these models with varying prediction steps and parameter sizes. The results indicate that, under limited data and parameters, the model performance steadily declines as the prediction step increases (Figure S1A). On the other hand, as the number of model parameters increases, the performance improves exponentially (Figure S1B). In Figure S1C, we present the KL divergence values for four models (RNN, low-rank RNN, EI RNN, and dend PLRNN) during 10-step predictions. Furthermore, we observe that the choice of evaluation metric can significantly influence the results for the same model. For example, when using MSE as a measure, RNN outperforms dend-PLRNN, whereas, with KL divergence, RNN performs worse than dend-PLRNN (Figure S1D). This observation highlights the importance of evaluating surrogate brain models from multiple perspectives.

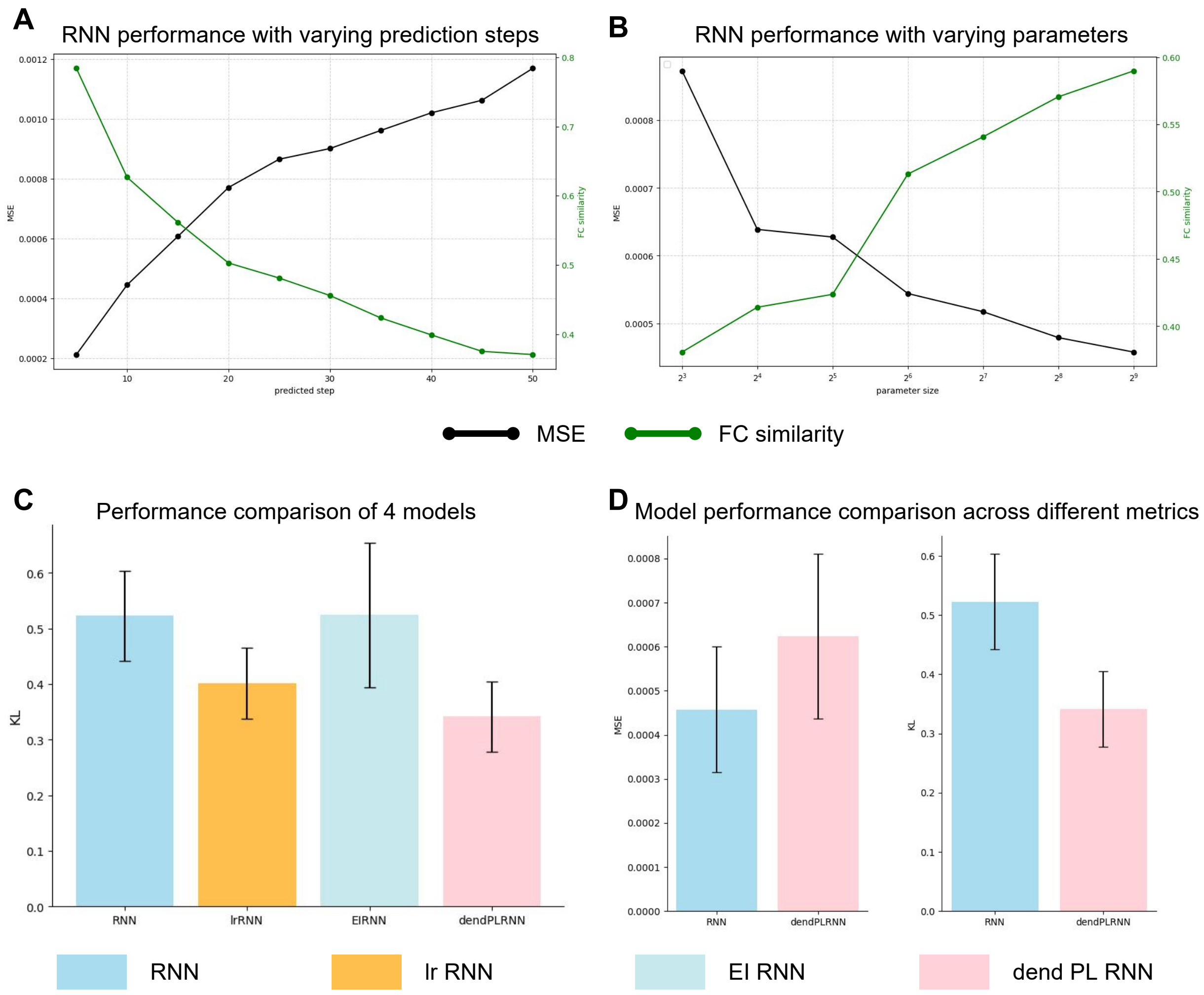

Figure S1: Benchmark and Evaluation of Surrogate Brain Models. **A** RNN performance with varying prediction steps: As the number of predicted steps increases, the model's performance steadily worsens measured by MSE and functional connectivity similarity. **B** RNN performance with varying parameters: Increasing the model parameters leads to an exponential improvement in performance, measured by MSE and functional connectivity similarity. **C** Performance comparison of four models (RNN, low-rank RNN, EI RNN, and dend PLRNN) based on KL divergence after 10-step predictions. **D** Model performance comparison across different evaluation metrics (MSE and KL divergence), showing that RNN outperforms dend-PLRNN with MSE but performs worse than dend-PLRNN when KL divergence is used. The data is from HUP123.

2 MATHEMATICAL BACKGROUND KNOWLEDGE

2.1 Well-posedness of Inverse Problem

The concept of "well-posedness" for inverse problems originates from the well-posedness theory proposed by the French mathematician Jacques Hadamard, which essentially judges whether the process of inferring model parameters from observational data has "good mathematical behavio". The detailed mathematical definition of well-posedness for inverse problems is as follows:

2.1.1 Basic Setup

Let an inverse problem be described by a *forward operator* $F : X \to Y$, where: X is the *parameter space* (usually a normed linear space, such as a Hilbert space or Banach space), and an element $x \in X$ represents the model parameter to be determined; Y is the *observation data space* (also a normed linear space), and an element $y \in Y$ represents the observed data; The operator F characterizes the mapping relationship of the forward problem: given a parameter x, data $y = F(x)$ is generated through the forward problem (e.g., physical laws, mathematical models).

The goal of the inverse problem is: given observational data y, find x such that $F(x) = y$.

2.1.2 Three Conditions for Well-posedness

An inverse problem is said to be *well-posed* if it satisfies the following three conditions:

1. **Existence** For "reasonable" observational data $y \in Y$ (typically practically observable data, which may belong to a subset $\mathcal{Y} \subseteq Y$), there exists at least one parameter $x \in X$ such that $F(x) = y$.

Mathematical formulation: $\forall y \in \mathcal{Y} \subseteq Y, \exists x \in X$ with $F(x) = y$.

2. **Uniqueness** For any two parameters $x_1, x_2 \in X$, if they generate the same observational data, then these two parameters must be equal.

Mathematical formulation: $\forall x_1, x_2 \in X$, if $F(x_1) = F(x_2)$, then $x_1 = x_2$.

(Note: Uniqueness ensures the "determinacy" of the solution, i.e., observational data does not correspond to multiple distinct model parameters.)

3. **Stability** The parameter x "continuously depends" on the observational data y: when the observational data y undergoes a small perturbation, the corresponding parameter x also undergoes only a small perturbation.

Mathematical formulation: From existence and uniqueness, we can define the inverse operator $F^{-1} : \mathcal{Y} \to X$ (i.e., $x = F^{-1}(y)$), and F^{-1} is a *continuous operator*. That is: $\forall \varepsilon > 0, \exists \delta > 0$ such that for any $y_1, y_2 \in \mathcal{Y}$, if $\|y_1 - y_2\|_Y < \delta$, then $\|F^{-1}(y_1) - F^{-1}(y_2)\|_X < \varepsilon$, where $\|\cdot\|_X$ and $\|\cdot\|_Y$ are norms on X and Y (used to measure "small perturbations"), respectively.

(Note: Stability is the most critical condition in well-posedness, ensuring that noise in observa-

tional data (inevitable errors) does not lead to "severe distortion" of the solution.)

2.2 Introduction to Optimization Methods

The core challenge in solving inverse problems for dynamical systems lies in identifying optimal model parameters. Once the objective function is determined, this problem transforms into minimizing the objective functional. Now we present optimization algorithms by progressively increasing complexity: starting from basic scenarios with differentiable objectives, moderate dimensionality, and no constraints; then advancing to constrained, high-dimensional, large-scale, and non-smooth problems.

For differentiable objective functions with moderate dimensionality (hundreds to thousands of dimensions) and no constraints, the standard approach Gradient Descent (GD) offers stability but slow convergence. Newton's method converges faster but is sensitive to ill-conditioned Hessians. The Levenberg-Marquardt algorithm balances these approaches through a damping parameter, combining stability with rapid convergence.

Neuroscience parameters often carry biological constraints such as non-negative connection strengths. Common solution strategies include penalty methods that convert constraints like $g(x) \leq 0$ to penalty terms $\lambda \max(0, g(x))^2$; sequential quadratic programming (SQP) that iteratively solves quadratic approximations with linearized constraints; and interior point methods that embed constraints via logarithmic barrier functions:

$$\min_{x} f(x) - \mu \sum \ln(-g(x)) \tag{11}$$

By gradually reducing $\mu > 0$, solutions remain feasible while converging to optimality with polynomial complexity.

Neuroscience models face dual challenges of high dimensionality and massive datasets. For high-dimensional problems, L-BFGS approximates Hessian inverse using limited vector pairs ($m \sim 10$) :

$$H_{k+1} = (I - \rho_k s_k y_k^T) H_k (I - \rho_k y_k s_k^T) + \rho_k s_k s_k^T \tag{12}$$

with $s_k = x_{k+1} - x_k$, $y_k = \nabla f(x_{k+1}) - \nabla f(x_k)$, reducing complexity to $\mathcal{O}(mn)$. For large-scale data, stochastic gradient descent (SGD) uses mini-batch gradients, enabling escape from local minima. The Adam optimizer combines momentum and adaptive learning via gradient moments, excelling in non-convex problems like neural activity modeling and sparse data scenarios.

When objectives involve sparsity regularization, discrete operations, or non-differentiable components such as in spiking neural networks, options include the gradient-free Nelder-Mead simplex method for low-dimensional problems and surrogate gradients that approximate gradients for non-differentiable functions, maintaining gradient-based efficiency while handling discontinuities in

high-dimensional systems.

Most algorithms require careful step size control. The Wolfe conditions ensure sufficient decrease and curvature, avoiding expensive exact line searches while preventing divergent updates.

$$f(x_k + \alpha d_k) \le f(x_k) + c_1 \alpha \nabla f(x_k)^T d_k \tag{13}$$

$$\nabla f(x_k + \alpha d_k)^T d_k \ge c_2 \nabla f(x_k)^T d_k \quad (0 < c_1 < c_2 < 1) \tag{14}$$

While line search methods are efficient but potentially divergent with poor step sizes, trust region methods offer an alternative approach. Their core principle involves approximating the objective function with a local quadratic model:

$$m_k(s) = f(x_k) + \nabla f(x_k)^T s + \frac{1}{2} s^T B_k s$$

where B_k approximates the Hessian. The step s is constrained by $\|s\| \le \Delta_k$. The ratio $\rho_k = \frac{f(x_k) - f(x_k + s_k)}{m_k(0) - m_k(s_k)}$ determines trust region adjustments: expand radius if $\rho_k \approx 1$ (good agreement), contract radius if ρ_k is small (poor agreement), or maintain radius otherwise. By simultaneously updating step size and direction, trust region methods better manage risks in non-convex, nonlinear optimization.

Termination conditions for optimization algorithms include gradient-based criteria $\|\nabla f(x_k)\| \le \epsilon$ that satisfy first-order optimality for smooth functions; function-value criteria $|f(x_{k+1}) - f(x_k)| \le \epsilon$ that are gradient-free and suitable for non-smooth objectives; parameter-change criteria $\|x_{k+1} - x_k\| \le \epsilon$ effective for low-dimensional problems with physical interpretations; and hybrid criteria with iteration limits that dominate in large-scale/stochastic optimization like deep learning.

The discussed optimization algorithms exhibit diverse characteristics and applications. Selecting appropriate methods for model parameter estimation depends on specific features of the inverse problem and data types in dynamical systems. For non-convex optimization challenges or scenarios with multiple local minima, combining different algorithms and strategies is often necessary to achieve reliable results.

3 SUPPLEMENTARY TABLE

3.1 Neural Mass Model

Table S1: Overview of Neural Mass Models

Neural Mass Model	Model Description	Platform
**Wilson–Cowan Model**	A neural mass model consisting of two state variables representing interacting excitatory and inhibitory neuronal populations, used to simulate the membrane potential dynamics of neural populations [6]. It can be extended to a neural field model by introducing a spatial convolution kernel, enabling the modeling of spatiotemporal propagation [7].	Neuralib, TVB, BrainPy
**Epileptor**	A neural mass model consisting of five state variables, representing two fast subsystems for epileptic discharges, one slow subsystem for slow-wave energy, and a slow time-scale variable. It simulates the onset, propagation, and termination of epileptic seizures [8, 9]. By incorporating a spatial convolution kernel, it can be extended to a neural field model for the simulation of spatial seizure spread [10].	TVB, BrainPy
**Jansen–Rit Model**	A neural mass model consisting of three state variables representing cortical pyramidal cells, excitatory interneurons, and inhibitory interneurons. It models the electrophysiological activity of cortical columns and can generate EEG-like signals [11, 12].	TVB, BrainPy
**Larter–Breakspear Model**	A neural mass model consisting of three state variables: the membrane potential of excitatory pyramidal cells, the membrane potential of inhibitory interneurons, and the proportion of open potassium ion channels. It captures the effects of extracellular potassium ion diffusion on coupling between neural populations and is used to investigate synchronous oscillations and their dynamics [13, 14].	TVB
**Thalamocortical Model**	A neural mass model consisting of six state variables describing thalamic excitatory neurons, thalamic inhibitory (reticular) neurons, and cortical excitatory neurons, each with a membrane potential and an associated gating variable. It models the generation of slow waves, spindles, and K-complexes, and reproduces EEG features observed during NREM sleep [15].	Neurolib, BrainPy

3.2 Comparison of White-, Black-, and Grey-box Approaches

	White-box	Black-box	Grey-box
**Tractability**	**High**<br>Parameters with physical meaning enable priors and data assimilation; mature solvers; identifiability typically stronger.	**Low–Moderate**<br>High dimensionality /parameterisation; weaker identifiability; data-hungry; uncertainty quantification is harder.	**Moderate**<br>Requires balancing constraints and flexibility; tractable via regularisation /priors or physics-informed losses.
**Expressivity**	**Moderate**<br>Flexibility constrained by mechanistic structure.	**Very high**<br>Few restrictions on function class; universal approximation.	**High**<br>Learnable modules augment mechanistic backbones.
**Interpretability**	**High**<br>Parameters/structures map to physiology.	**Low**<br>Parameters lack physiological meaning.	**Moderate**<br>Constrained structures remain interpretable.
**Falsifiability**	**High**<br>Clear mechanistic hypotheses enable direct experimental falsification.	**Low**<br>Indirect via prediction accuracy (no explicit mechanistic hypotheses), yet less compelling due to high flexibility.	**Moderate**<br>Hybrid architectures combine biological priors and data-driven approaches, supporting partial hypothesis validation.
**Examples**	NMMs (Wilson-Cowan, Jansen-Rit); NFM (Robinson model), connectome-based BNMs.	RNN/LSTM/GRU; Neural ODE/SDE; Transformers; VAEs; Mamba.	SNNs; LNNs; PINNs; HNNs; ANNs with priors.

Table S2: Transposed comparison of white-box, black-box, and grey-box approaches along tractability, expressivity, interpretability, and falsifiability, with representative examples.

References

[1] Lohith G Kini, John M Bernabei, Fadi Mikhail, Peter Hadar, Preya Shah, Ankit N Khambhati, Kelly Oechsel, Ryan Archer, Jacqueline Boccanfuso, Erin Conrad, et al. Virtual resection predicts surgical outcome for drug-resistant epilepsy. *Brain*, 142(12):3892–3905, 2019.

[2] John M Bernabei, Nishant Sinha, T Campbell Arnold, Erin Conrad, Ian Ong, Akash R Pattnaik, Joel M Stein, Russell T Shinohara, Timothy H Lucas, Dani S Bassett, et al. Normative intracranial eeg maps epileptogenic tissues in focal epilepsy. *Brain*, 145(6):1949–1961, 2022.

[3] Julia Costacurta, Shaunak Bhandarkar, David Zoltowski, and Scott Linderman. Structured flexibility in recurrent neural networks via neuromodulation. *Advances in Neural Information Processing Systems*, 37:1954–1972, 2024.

[4] H Francis Song, Guangyu R Yang, and Xiao-Jing Wang. Training excitatory-inhibitory recurrent neural networks for cognitive tasks: a simple and flexible framework. *PLoS computational biology*, 12(2):e1004792, 2016.

[5] Manuel Brenner, Florian Hess, Jonas M Mikhaeil, Leonard F Bereska, Zahra Monfared, Po-Chen Kuo, and Daniel Durstewitz. Tractable dendritic rnns for reconstructing nonlinear dynamical systems. In *International conference on machine learning*, pages 2292–2320. Pmlr, 2022.

[6] Hugh R Wilson and Jack D Cowan. Excitatory and inhibitory interactions in localized populations of model neurons. *Biophysical journal*, 12(1):1–24, 1972.

[7] Hugh R Wilson and Jack D Cowan. A mathematical theory of the functional dynamics of cortical and thalamic nervous tissue. *Kybernetik*, 13(2):55–80, 1973.

[8] Viktor K Jirsa, William C Stacey, Pascale P Quilichini, Anton I Ivanov, and Christophe Bernard. On the nature of seizure dynamics. *Brain*, 137(8):2210–2230, 2014.

[9] Viktor K Jirsa, Timothée Proix, Dionysios Perdikis, Michael Marmaduke Woodman, Huifang Wang, Jorge Gonzalez-Martinez, Christophe Bernard, Christian Bénar, Maxime Guye, Patrick Chauvel, et al. The virtual epileptic patient: individualized whole-brain models of epilepsy spread. *Neuroimage*, 145:377–388, 2017.

[10] Timothée Proix, Viktor K Jirsa, Fabrice Bartolomei, Maxime Guye, and Wilson Truccolo. Predicting the spatiotemporal diversity of seizure propagation and termination in human focal epilepsy. *Nat Commun*, 9(1):1088, 2018.

[11] Ben H Jansen and Vincent G Rit. Electroencephalogram and visual evoked potential generation in a mathematical model of coupled cortical columns. *Biological cybernetics*, 73(4):357–366, 1995.

[12] Ben H Jansen, George Zouridakis, and Michael E Brandt. A neurophysiologically-based mathematical model of flash visual evoked potentials. *Biological cybernetics*, 68:275–283, 1993.

[13] Raima Larter, Brent Speelman, and Robert M Worth. A coupled ordinary differential equation lattice model for the simulation of epileptic seizures. *Chaos: An Interdisciplinary Journal of Nonlinear Science*, 9(3):795–804, 1999.

[14] Michael Breakspear, John R Terry, and Karl J Friston. Modulation of excitatory synaptic coupling facilitates synchronization and complex dynamics in a nonlinear model of neuronal dynamics. *Neurocomputing*, 52:151–158, 2003.

[15] Michael Schellenberger Costa, Arne Weigenand, Hong-Viet V Ngo, Lisa Marshall, Jan Born, Thomas Martinetz, and Jens Christian Claussen. A thalamocortical neural mass model of the eeg during nrem sleep and its response to auditory stimulation. *PLoS computational biology*, 12(9):e1005022, 2016.
